# From Plant Metabolites to Pollinator Safety: Rethinking Selectivity of Botanical Insecticides in Bees—A Review

**DOI:** 10.3390/biology15120948

**Published:** 2026-06-17

**Authors:** Silvana Aparecida de Souza, Isabella Maria Pompeu Monteiro Padial, José Vinícius Conceição da Cruz, Matheus Gonçalves Camargo, Marcia Regina Faita, Rosilda Mara Mussury

**Affiliations:** 1 Postgraduate Program in Entomology and Biodiversity Conservation, Faculty of Biological and Environmental Sciences, Federal University of Grande Dourados, Dourados–Itahum Highway, km 12, Dourados 79804-970, MS, Brazil; silvanaadesouza@gmail.com (S.A.d.S.); bellapadial@hotmail.com (I.M.P.M.P.); 2 Undergraduate Program in Biological Sciences, Faculty of Biological and Environmental Sciences, Federal University of Grande Dourados, Dourados–Itahum Highway, km 12, Dourados 79804-970, MS, Brazil; jose.vexu@gmail.com; 3 Undergraduate Program in Biotechnology, Faculty of Biological and Environmental Sciences, Federal University of Grande Dourados, Dourados–Itahum Highway, km 12, Dourados 79804-970, MS, Brazil; camargomatheus6@gmail.com; 4 Department of Entomology, Center for Biological and Health Sciences, Federal University of Viçosa, Peter Henry Rolfs Avenue, University Campus, Vicosa 36570-900, MG, Brazil; marcia.faita@ufv.br

**Keywords:** bees, sublethal effects, risk assessment, Integrated Pest Management, botanical biopesticides, ecotoxicology, non-target organisms

## Abstract

Botanical insecticides have re-emerged as promising tools within Integrated Pest Management (IPM) due to their biodegradability, chemical diversity, and potential compatibility with resistance management strategies. Although frequently considered safer alternatives to synthetic pesticides, growing evidence indicates that these compounds may also affect non-target organisms, particularly bees. This review discusses the selectivity of botanical insecticides toward pollinators by integrating historical perspectives, mechanisms of action, ecotoxicological effects, and current limitations in risk assessment approaches. Botanical insecticides may induce both lethal and sublethal effects, including alterations in behavior, locomotion, feeding, development, reproduction, and physiology across different bee groups. We also demonstrate that most available studies remain concentrated on *Apis mellifera*, adult workers, and acute laboratory assays, while important pollinator groups and chronic exposure scenarios remain poorly explored. Furthermore, current regulatory protocols are still largely based on models developed for synthetic pesticides. Expanding ecotoxicological approaches is therefore essential to improve pollinator safety assessments and support more sustainable pest management strategies.

## Simple Summary

Botanical insecticides are natural plant-derived products whose insecticidal activity results from the synergistic action of secondary metabolites capable of affecting the biological traits of insect pests. Although these compounds are frequently associated with low environmental and human toxicity, important gaps remain regarding their effects on beneficial organisms, particularly bees. In this review, we discuss how botanical insecticides may affect different bee groups, not only by causing mortality, but also by inducing behavioral, physiological, developmental, and reproductive alterations. Such effects may compromise essential activities for colony survival and maintenance, including foraging and reproduction. We also demonstrate that most currently available protocols and studies remain largely restricted to laboratory assays using *Apis mellifera*, limiting the extrapolation of results to other bee groups, such as stingless bees, bumblebees, and solitary bees. Therefore, we highlight the need for broader assessment approaches capable of considering pollinator diversity and realistic exposure conditions in agricultural environments.

## 1. Introduction

Bees represent the primary group of pollinators and play a central role in agriculture, being responsible for the pollination of approximately 78.9% of major agricultural crops [1,2,3]. The pollination services provided by these insects contribute both to biodiversity conservation and agricultural productivity by increasing the quantity and quality of fruits and seeds and promoting more sustainable food production systems [4,5].

However, insecticides, in general, can affect bee performance, behavior, ecology, and communication [6,7,8]. Agrochemicals, particularly herbicides and insecticides, have been associated with a variety of adverse effects on these pollinators, including alterations in growth, fecundity, longevity, and behavior [6], as well as reductions in wax and honey production [9], among other sublethal effects. Concern regarding these impacts has intensified over recent decades due to the growing body of evidence linking pesticide exposure to bee population declines and the disruption of ecological interactions within agroecosystems [6,7,8].

In this context, increasing restrictions on the use of synthetic insecticides due to their toxicity and environmental persistence [10], together with the rising costs and challenges associated with the development of new classes of compounds [11,12,13,14], have stimulated the adoption of Integrated Pest Management (IPM) and renewed interest in botanical insecticides as more selective, less persistent, and resistance-management-compatible alternatives [15,16,17,18].

Botanical insecticides are derived from plant secondary metabolites and may contain compounds such as alkaloids, terpenes, and flavonoids capable of affecting insect biology through multiple mechanisms of action [19,20,21]. These compounds can induce feeding deterrence, developmental impairment, reproductive alterations, behavioral modifications, and mortality in pest insects [20,21]. However, although these compounds are often regarded as safer and more environmentally compatible alternatives, their selectivity toward non-target organisms, particularly pollinators, remains incompletely understood [15,16,17,18].

Despite this perception, recent reviews addressing botanical insecticides, biopesticides, pesticide risk, and pollinator exposure have shown that the assumption that botanical insecticides are inherently safe for non-target organisms remains insufficiently supported by empirical evidence [22,23,24,25,26]. A growing number of studies indicate that plant-derived compounds may also affect beneficial arthropods, including bees, causing lethal and sublethal effects that vary according to the compounds involved, exposure routes, developmental stage, and species evaluated. Furthermore, the available knowledge regarding the selectivity of botanical insecticides toward bees remains fragmented and unevenly distributed across different taxonomic groups [22,23,25]. Consequently, significant uncertainties persist regarding the selectivity of these compounds and the ability of currently available evidence to support reliable safety assessments for bees. Furthermore, there is still no comprehensive synthesis that simultaneously integrates the different bee groups, types of botanical compounds, exposure routes, and categories of effects reported in the literature.

Therefore, rather than merely compiling toxicological studies, this review adopts a holistic perspective to critically examine the current state of knowledge concerning interactions between botanical insecticides and bees. We synthesize evidence involving different bee groups, developmental stages, exposure routes, and categories of effects, aiming to identify recurring patterns, methodological biases, and important knowledge gaps. In addition, we discuss the limitations of current risk assessment models and propose research priorities and methodological improvements necessary to generate more robust and ecologically relevant evaluations of the safety of botanical insecticides for bees within the context of Integrated Pest Management.

## 2. Materials and Methods

A structured literature review was conducted focusing on the toxicity and safety of botanical insecticides to bees within the context of Integrated Pest Management (IPM). Bibliographic searches were performed using Scopus, Web of Science Core Collection, PubMed, and Google Scholar databases. Searches were conducted using combinations of descriptors related to botanical insecticides, bees, and toxicological assessment. The following terms were used: “botanical insecticides”, “botanical biopesticides”, “plant-derived insecticides”, “plant extracts”, “essential oils”, “secondary metabolites”, “bees”, “pollinators”, “*Apis mellifera*”, “stingless bees”, “bumblebees”, “solitary bees”, “toxicity”, “selectivity”, “risk assessment”, “acute toxicity”, “chronic toxicity”, “sublethal effects”, “behavioral effects”, “developmental effects”, “reproductive effects”, “pesticide exposure”, and “OECD guidelines”.

The literature search included studies published from 1930 to 2026, corresponding to the period between the earliest identified publications related to the topic and the completion of this review. Articles published in English, Portuguese, French and Spanish were considered. In addition, a bibliometric analysis was performed using the Bibliometrix package (version 5.4.0) in the R environment. Data were retrieved from the Scopus database using the following search strategy: (“botanical insecticide” OR “botanical pesticide” OR “botanical biopesticide” OR “essential oil”) AND (“bee” OR “*Apis mellifera*” OR “bumblebee” OR “stingless bee” OR “Meliponini” OR “*Osmia*”). The search covered all years indexed in the database up to 2026 and included only documents relevant to the scope of this review. Bibliometric indicators were calculated, including annual scientific production, cumulative publication growth, and mean citations per article according to the year of publication. Additional references were identified through manual screening of the reference lists of selected studies.

The figures were created by the authors using Canva Pty Ltd. (2026), Microsoft PowerPoint (version 16.0), and Bibliometrix (version 5.4.0). In some cases, artificial intelligence tools were used solely to assist with the visual refinement and final presentation of the figures, without generating or modifying the scientific data, interpretations, or conclusions. All figures were reviewed and edited by the authors. This study consists of a narrative rather than a systematic review, aiming to integrate current evidence regarding the toxicity of botanical insecticides across different bee groups.

## 3. Historical Development of Research on the Effects of Botanical Insecticides on Bees

The first scientific observations regarding the toxic effects of chemical substances on non-target organisms emerged during the early decades of the twentieth century, long before the formal development of ecotoxicology, and largely originated from field studies investigating unintended consequences of chemical pest control [27,28,29,30,31,32,33].

Among these pioneering studies, the work conducted by Woglum et al. [31] stands out. The authors investigated citrus orchards in California between 1907 and 1947 and demonstrated that insecticide applications, even under relatively low-intensity management regimes, could interfere with natural enemy populations, affecting biological control processes and altering pest dynamics. Initially, under systems with low application frequency, the combination of chemical control and natural enemies appeared effective, with relatively limited impacts on parasitoids and predators. However, with the intensification of insecticide use, particularly following the introduction of new compounds and more frequent spraying programs, significant reductions in beneficial organisms occurred, resulting in ecological imbalances and secondary pest outbreaks.

Studies such as these represented some of the earliest indications that pest control practices could disrupt ecological interactions within agricultural systems [34]. Thus, by the early 1950s, it was already understood that intensive use of available insecticides could affect organisms beyond target pest species [32,35]. Within this context, bees gradually became incorporated into toxicological studies, initially due to their agricultural importance as pollinators.

During the 1940s and 1950s, the first experimental investigations into pesticide toxicity in bees focused primarily on acute lethal effects, reflecting both the methodological limitations and the applied research priorities of that period. Experimental studies demonstrated that insecticide and fungicide applications under laboratory and field conditions could cause direct intoxication, repellency, and alterations in bee foraging behavior [36,37,38].

In this context, the study conducted by Butler et al. [36] deserves particular attention as one of the first systematic investigations evaluating the effects of insecticide and fungicide sprays on *Apis mellifera* (Hymenoptera: Apidae) (Linnaeus), integrating both laboratory and field approaches. In their experiments, the authors employed controlled feeding assays to simultaneously evaluate toxicity and compound acceptability, allowing the distinction between lethal and behavioral effects. Importantly, the study highlighted two aspects that remain highly relevant today. First, it provided detailed descriptions of intoxication symptoms, including paralysis, tremors, and crawling behavior, demonstrating that pesticide effects extend beyond immediate lethality. Second, it identified multiple exposure routes, emphasizing contaminated water collection as the primary route of intoxication and pollen as a secondary source capable of generating prolonged effects within colonies.

From the 1970s onward, the understanding of pesticide effects on bees and other non-target organisms became considerably more complex as experimental advances demonstrated that pesticide impacts extend far beyond acute mortality, encompassing a broad range of sublethal and ecological effects [8,39,40,41].

Evidence showed that exposure to low concentrations of chemical compounds may impair complex behavioral functions, such as communication and orientation during foraging, while simultaneously allowing the continuous collection of contaminated resources and the progressive accumulation of toxins within the organism. At the same time, certain compounds were found to selectively affect different developmental stages, causing significant impairments to brood development even in the absence of apparent effects on adult individuals [8,39,40,41].

Nevertheless, acute and chronic contamination of producers, consumers, and non-target organisms resulting from synthetic pesticide use became increasingly common over the following decades. Environmental degradation, disruption of pollination and biological control interactions, and the evolution of pesticide resistance became major concerns by the end of the twentieth century [42,43,44,45,46].

**Figure 1:**
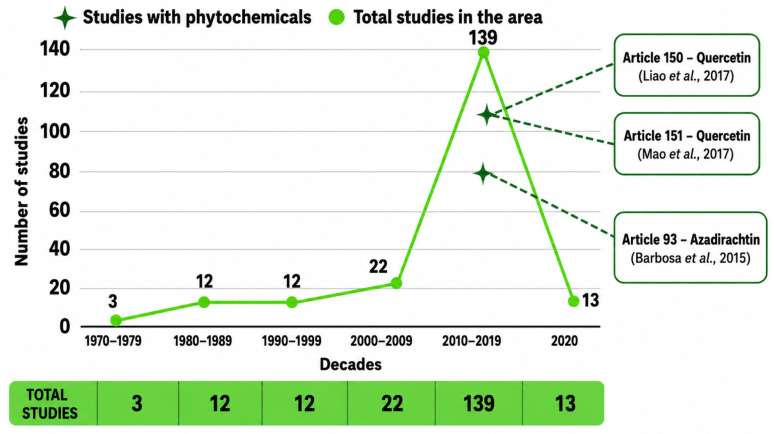
Temporal distribution of compiled studies evaluating the effects of pesticides on bees across decades. The green line represents the total number of studies published per decade, while highlighted markers indicate studies specifically involving phytochemicals. The highlighted studies correspond to [47,48,49]. Adapted from Rossi et al. [8].

It is noteworthy that, even after recognition that chemical pest control could negatively affect non-target organisms, particularly bees, the response from both the scientific community and the market remained relatively delayed. This pattern is evidenced by works such as *Abelhas & Agrotóxicos*: *Compilação sobre as evidências científicas dos impactos dos agrotóxicos sobre as abelhas* by Rossi et al. [8], which compiled available studies addressing pesticide toxicity in bees over time. Although the compilation includes studies dating back to the 1970s, the authors themselves highlight the exponential increase in scientific production only in recent years, with the 2010s alone accounting for approximately 69% of all studies included in the compilation (Figure 1).

**Figure 2:**
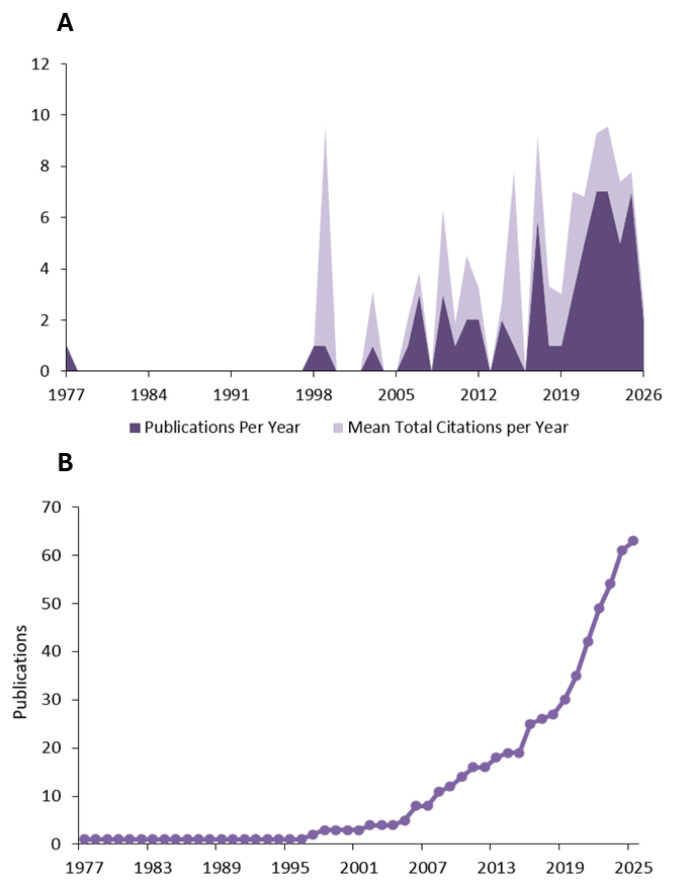
Bibliometric analysis of the literature on botanical insecticides and bees retrieved from the Scopus database in June 2026. The search query applied to titles, abstracts, and keywords was (“botanical insecticide” OR “botanical pesticide” OR “botanical biopesticide” OR “essential oil”) AND (“bee” OR “*Apis mellifera*” OR “bumblebee” OR “stingless bee” OR “Meliponini” OR “*Osmia*”). (**A**) Annual scientific production and mean citations per article according to publication year. (**B**) Cumulative growth in the number of publications over the analyzed period. Figure prepared by the authors.

This scenario stimulated the emergence of concepts such as Integrated Pest Management (IPM) [16], together with the revival of alternative control strategies, including botanical insecticides, which regained scientific attention [17]. Consequently, the impact generated by the negative consequences associated with synthetic insecticide use can be observed through the shift in research trends from the beginning of the twenty-first century onward. For instance, Isman and Grieneisen [17] conducted a bibliometric analysis of publications involving botanical insecticides and demonstrated that studies within the botanical insecticide field increased from 2% to 21% between 1980 and 2011 (Figure 2).

The trend identified by Isman and Grieneisen [17] appears to have continued in subsequent years. To complement these findings, a bibliometric analysis was conducted based on publications retrieved from the Scopus database. The results indicate a sustained increase in scientific production related to botanical insecticides and bees, particularly after the 2000s, with accelerated growth during the last decade (Figure 2). The cumulative increase in the number of publications, together with rising citation rates, reflects the growing scientific interest in understanding not only the efficacy of botanical insecticides but also their potential impacts on pollinators. This pattern highlights the progressive incorporation of pollinator safety into discussions concerning the sustainable use of botanical pesticides.

Similarly to what occurred with synthetic pesticides, the evaluation of the effects of botanical insecticides on bees also developed slowly and may even be considered an even more delayed process. The initial assumption that plant-derived compounds were inherently safe for pollinators limited the development of ecotoxicological studies in this field [22,50]. Nevertheless, evidence accumulated over time demonstrated that secondary metabolites present in nectar and pollen may affect bee survival and behavior, indicating that these organisms are not necessarily insensitive to plant chemical defenses. These findings contradict the assumption that “natural” compounds are automatically safe and reinforce the need for more rigorous risk assessment approaches [22,51,52].

Thus, it is not coincidental that studies addressing the toxicity of botanical insecticides to bees also increased substantially from the 2010s onward. As shown in Figure 2, this increase occurred only after a period in which the broader literature on botanical insecticides had already undergone considerable expansion, indicating that investigations into their effects on pollinators emerged only after a large body of more general data had already been generated. In addition, the compilation conducted by Catania et al. [22], encompassing studies published between 1930 and 2022, demonstrates that most research involving bees has been concentrated in recent decades, reinforcing the existence of a temporal mismatch between the development of these compounds and the assessment of their impacts on non-target organisms.

Despite these advances, current knowledge remains limited when compared to that available for synthetic pesticides. This difference becomes evident when comparing the number of studies compiled by Rossi et al. [8] and Catania et al. [22], which reported 198 studies on synthetic pesticides versus only 54 studies on botanical insecticides, despite the latter having been evaluated for nearly forty fewer years. Recent reviews further emphasize that studies investigating botanical insecticides in bees are still scarce, frequently restricted to a few model species, and lack standardized methodologies for risk assessment [22,50].

Overall, the historical development of pesticide research reveals a gradual transition from approaches focused exclusively on acute toxicity toward broader perspectives incorporating sublethal effects, multiple exposure routes, and ecological consequences. In the case of botanical insecticides, however, this process was more delayed and less explored, reflecting both the historically limited attention given to these products and the persistent assumption of their intrinsic safety, which contributed to existing gaps in understanding their effects on bees.

## 4. Selectivity and Impacts on Bees

### 4.1. Importance of Pollinators

Pollinators play a central role in the reproduction of a large proportion of angiosperms [52,53] and in maintaining the structure and sustainability of ecosystems [54], constituting one of the most important components of plant–animal interactions [54]. Recent estimates indicate that approximately 89.5% of angiosperm species depend, to some extent, on animal-mediated pollination [55].

Among pollinators, bees represent the most abundant group and are essential for agriculture, contributing to the pollination of approximately 78.9% of major crop species [3,56]. The decline in pollination services may result in estimated agricultural yield losses ranging from 3% to 8%, in addition to compromising other production-related parameters. Crops such as cotton, soybean, sunflower, strawberry, grapevine, and tomato show increased yield and quality when associated with pollination services [3], whereas crops such as apple and melon rely heavily on insect pollination for adequate fruit set [56,57].

In addition to directly influencing the productivity and quality of numerous crops, bees support production chains associated with beekeeping and meliponiculture through the production of honey, wax, propolis, pollen, and other commercially valuable bee products. In this context, pollinator conservation extends beyond biodiversity protection and is directly linked to food security, agricultural stability, and the promotion of sustainable livelihoods. These contributions are closely associated with several of the United Nations Sustainable Development Goals (SDGs), particularly those related to zero hunger, sustainable production, and biodiversity conservation [58]. Consequently, the impairment of pollination services may have negative consequences for food security and human health, including risks associated with malnutrition [59].

Despite their ecological and economic importance, bee populations have experienced well-documented declines in recent decades [60,61]. This scenario has been associated with the combined action of multiple factors, including habitat loss and fragmentation, expansion of monoculture systems, climate change, and continuous exposure to pesticides during foraging activities, with contaminants potentially remaining stored within colonies for extended periods [41,62,63]. From this perspective, evaluating insecticide selectivity becomes essential to reconcile pest management practices with the conservation of non-target organisms.

### 4.2. Current Risk Assessment Approaches

An ideal insecticide should act specifically against the target pest while minimizing adverse effects on non-target organisms. However, absolute selectivity is difficult to achieve, as many insecticides may affect beneficial organisms and pose risks to human health [64,65]. In this context, selectivity assessments must consider that insects can be exposed to pesticides through multiple routes. In bees, contamination may occur through the ingestion of contaminated food or water, contact with nesting materials, indirect contact with residues present within the colony [66], the inhalation of airborne particles, or direct contact with pesticide residues deposited on plant surfaces [67].

Environmental risk assessments have been used to estimate the likelihood that pesticides may compromise the survival of honey bee colonies [68]. Toxicity is evaluated according to the exposure route (acute or chronic) and the severity of the observed effects (lethal or sublethal). Acute toxicity is characterized by individual mortality occurring within 72 h after exposure, whereas chronic toxicity refers to long-term (sublethal) effects, such as physiological and/or behavioral alterations [65,69,70].

The need to evaluate these effects in a comparable and reproducible manner has led to the adoption of model species in ecotoxicological protocols designed to protect pollinators. Surrogate species therefore represent a methodological solution to the practical impossibility of testing the full diversity of organisms potentially exposed to plant protection products. In this context, regulatory risk assessment for plant protection products, including both synthetic and botanical insecticides, relies on *Apis mellifera* (Hymenoptera: Apidae) as a protective surrogate species in risk assessment studies [71,72]. The use of A. mellifera in ecotoxicological evaluations is directly related to its wide availability [73,74], ease of rearing, handling, and transport [71], year-round availability of individuals [75], capacity to provide sufficient numbers of individuals for laboratory assays, and good adaptation to controlled experimental conditions [76].

The Organization for Economic Co-operation and Development (OECD) plays a central role in this process by establishing internationally recognized protocols and recommendations with standardized methods for regulatory assessments of chemical and environmental safety [77]. These guidelines contribute to test standardization, result comparability, and data acceptance among countries participating in the Mutual Acceptance of Data (MAD) system, provided that studies are conducted according to OECD Test Guidelines and Good Laboratory Practice principles [78].

According to OECD guidelines, the toxicological assessment of plant protection products using *A. mellifera* can be conducted at different levels, including initial laboratory tests and complementary semi-field and field studies. At the first tier, acute oral toxicity [79] and acute contact toxicity [80], as well as protocols (Test Guidelines, TGs), constitute the basis of the initial assessment and are generally required for regulatory purposes. When necessary, these tests may be complemented by single-exposure acute toxicity assays in immature stages [81], and chronic exposure studies in adult bees [82].

At a second tier, more flexible guidance documents (GDs) include laboratory assays such as repeated larval exposure tests [83] as well as semi-field and field studies, including brood development assessments under semi-field conditions [84] and homing flight tests evaluating the return of foragers after sublethal oral exposure [85]. For non-*Apis* species, recommendations are available for acute laboratory tests in adult Bombus workers, including both contact and oral exposure routes [86,87]. For *Osmia*, only one guideline is currently available [88], which addresses acute laboratory contact toxicity tests using adult females (Table 1) [79,80,81,82,83,84,85,86,87,88]. The OECD guidelines and guidance documents listed in Table 1 are not specific to biopesticides or botanical insecticides but rather apply broadly to the toxicological assessment of plant protection products in bees. Therefore, in the absence of protocols specifically designed for botanical insecticides, these guidelines are commonly used as the reference framework for evaluating their potential effects on pollinators. No specific protocols were identified for bee species belonging to the tribe Meliponini.

The OECD guidelines for pesticide assessment in pollinators include laboratory assays with *A. mellifera*, *Bombus* spp., and *Osmia* spp., covering oral exposure, topical contact, larval toxicity, chronic toxicity, and acute toxicity in adults. However, guidance documents addressing more complex endpoints, such as brood development under semi-field conditions, repeated larval exposure, and homing flight after sublethal exposure, remain centered on *A. mellifera*. Consequently, the predominance of *A. mellifera* in ecotoxicological testing has established a paradigm in which a single eusocial species is used to represent the extensive diversity of bees, estimated at approximately 20,000 species worldwide [89]. Although operationally useful, this approach may overlook risks faced by other bee groups, particularly given the considerable variation in morphology, physiology, life-history traits, nesting biology, foraging behavior, and exposure pathways that can influence susceptibility to insecticides.

### 4.3. Lethal and Sublethal Effects of Botanical Insecticides on Bees

Synthetic insecticides are widely recognized for their toxicity to beneficial organisms [6] and their greater environmental persistence [90,91], whereas botanical insecticides have emerged as promising alternatives for ecologically based pest management. This is largely due to their diverse modes of action, biodegradability, and, in many cases, lower impacts on non-target organisms and human health [92,93]. Nevertheless, botanical insecticides can also cause lethal and sublethal effects in bees [22], highlighting the need for toxicological assessments of plant-derived insecticides in this group.

Considering the chemical complexity of botanical insecticides and the potential for synergistic interactions among secondary metabolites [94], the toxicity of these products should not be interpreted solely as the consequence of exposure to a single active molecule. Essential oils, plant extracts, and isolated compounds may differ in chemical composition, concentration, volatility, persistence, and mode of action, all of which directly influence the type and magnitude of effects observed in bees. Consequently, responses such as mortality, reduced locomotion, feeding deterrence, impaired foraging, and reproductive or developmental alterations may be associated with different mechanisms, including neurotoxicity, metabolic interference, antifeedant activity, or growth regulation.

**Figure 3:**
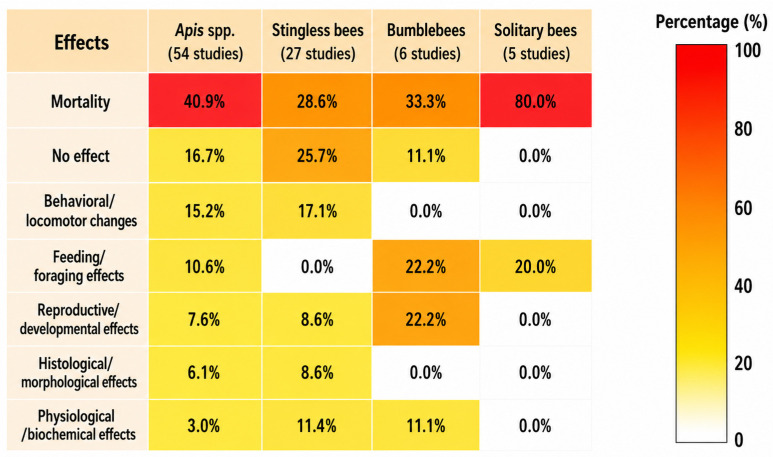
Distribution of lethal and sublethal effects of botanical insecticides reported for different bee groups. Values represent the percentage of records assigned to each effect category relative to the total number of studies analyzed for each group: *Apis* spp. (54 studies), stingless bees (27 studies), bumblebees (6 studies), and solitary bees (5 studies). The color scale indicates the relative frequency of each effect category, ranging from white (no records reported) to red (highest frequency). The category “No effect” indicates the absence of detectable effects in the parameters evaluated within a given study and should not be interpreted as evidence of overall safety. Data extracted and reorganized from Catania et al. [22]. Figure prepared by the authors.

Therefore, the toxicity of botanical insecticides should be evaluated not only in terms of their ability to cause mortality (lethal effects), whether immediately or after a given exposure period, but also as a range of alterations (sublethal effects) capable of impairing essential functions related to survival, reproduction, and population maintenance [95,96,97]. The physiological alterations associated with lethal and sublethal effects reported in *Apis* spp., stingless bees, bumblebees, and solitary bees are summarized in Figure 3.

Mortality remains the most frequently reported effect among different bee groups, accounting for an average of 45.7% of the records analyzed (Figure 3). This pattern indicates a tendency for studies to focus on acute toxicity tests, which may limit the detection of long-term effects. The occurrence of sublethal effects across multiple categories suggests that immediate survival should not be interpreted as evidence of the absence of biological impacts. Furthermore, the uneven distribution of reported effects demonstrates that toxicity studies on botanical insecticides remain heavily focused on the model species *A. mellifera*, neglecting other bee taxa and limiting our understanding of effects on stingless bees, bumblebees, and solitary bees.

Mortality caused by botanical insecticides may occur at different developmental stages, affecting both immature and adult bees. For example, in *A. mellifera*, andiroba oil (*Carapa guianensis* Aublet, Meliaceae) and garlic extract (*Allium sativum* L., Amaryllidaceae) caused high larval mortality, whereas citronella extract (*Cymbopogon* sp., Poaceae) and eucalyptus oil (*Eucalyptus* sp., Myrtaceae) resulted in high mortality in adult bees [97]. Similarly, a product based on *Quassia amara* L. (Simaroubaceae) extract caused more than 80% mortality in the eggs and larvae of *Osmia cornuta* (Hymenoptera: Megachilidae) when applied at field rates [98]. These findings demonstrate that assessments focused solely on adult survival may underestimate damage to immature stages, thereby compromising the interpretation of effects on colony renewal or species persistence in the case of solitary bees. The ecological consequences of mortality extend beyond the numerical loss of individuals and depend directly on the social organization of the affected species. In eusocial species (*Apis* spp. and stingless bees of the tribe Meliponini), colony stability depends on division of labor and overlapping generations, with younger workers performing nest tasks and older workers carrying out foraging and colony defense activities [99,100]. Even in more flexible social systems, such as those of bumblebees, task cooperation remains essential [101]. Consequently, worker loss disrupts the internal functioning of the colony, impairing processes ranging from brood care to colony maintenance [102].

In contrast, the ecological impact is even more severe in solitary bees because no colony exists to buffer individual losses. A single female is responsible for nest construction, resource collection, oviposition, and nest defense [103,104]. Therefore, the death of a solitary female may directly interrupt reproduction, reducing offspring production and compromising lineage persistence. This highlights that toxicity studies based exclusively on social species may not adequately represent risks faced by solitary bees. When exposure does not result in immediate mortality, damage may manifest through sublethal effects. Behavioral alterations are particularly common, accounting for an average of 16.15% of the reported records and occurring mainly in *Apis* spp. and stingless bees (Figure 3). Among these responses, locomotor impairments deserve special attention because they may indicate neurobehavioral disruption, expressed as reduced motor coordination and movement capacity. Reduced walking speed and shorter travel distances, observed in *Trigona hyalinata* (Lepeletier, 1836) (Hymenoptera: Apidae) and *A. mellifera* exposed to the essential oils of *Thymus vulgaris* L. and *Origanum vulgare* L. (Lamiaceae) [105], illustrate a loss of motor coordination that interferes with the daily activities of bees. Within the nest, impaired locomotion may compromise critical tasks such as cell cleaning, food processing, brood care [106] and thermoregulation in brood areas [107,108].

By affecting locomotion, botanical insecticides may also directly interfere with feeding and foraging. The occurrence of these effects in *Apis* spp., bumblebees, and solitary bees indicates that sublethal impacts can directly compromise resource collection and food intake, given that foraging is closely linked to individual performance, colony functioning, and colony population dynamics [109]. Because this activity depends on the integration of sensory perception, learning, memory, and movement [110,111,112,113], affected individuals may require more time to recognize and locate food resources or may fail to return to the colony. Such declines in field activity have been documented for azadirachtin-based formulations (*Azadirachta indica* A. Juss., Meliaceae), which reduced the number of *A. mellifera* foragers in melon fields in Brazil [114] and of *Apis cerana indica* (Fabricius, 1798), *Apis dorsata* Fabricius, 1793, and *A. mellifera* in mustard crops in India [115].

In bumblebees (*Bombus terrestris* (Linnaeus, 1758); Hymenoptera: Apidae), sublethal doses of azadirachtin reduced pollen collection in legume fields [116,117,118], potentially compromising brood nutrition. In solitary bees of the genus *Osmia*, the impact is even more direct on reproductive success. Because females must provide each brood cell independently, any reduction in foraging efficiency directly decreases nest construction and brood provisioning rates, thereby reducing offspring production and limiting population recruitment [119,120].

The reduction in foraging activity disrupts the colony’s resource supply chain. A shortage of nectar limits the availability of essential energy resources [121,122], while reduced pollen intake restricts access to proteins, lipids, vitamins, and minerals that are indispensable for brood development and the nutrition of young adults [123,124]. Similarly, the scarcity of non-food resources, such as water, which is used for evaporative thermoregulation [125,126], and resins, which are essential for nest architecture and protection against pathogens and predators [127,128], may weaken the colony as a whole.

Although feeding deterrence and feeding suppression are desirable traits in pest management because they reduce plant damage, they may represent a serious risk to bees, whose survival depends on the continuous intake of resources required for flight, metabolism, and reproduction [121,127,128,129]. A clear example is the deterrent effect observed in *A. mellifera* workers exposed to syrup contaminated with essential oils of *Lippia sidoides* Cham. (Verbenaceae) [129]. Although no mortality was observed during the first 24 h of exposure, the marked reduction in food consumption demonstrates how sensitive feeding behavior can be, highlighting potential risks that may remain undetected when mortality is used as the sole endpoint.

**Figure 4:**
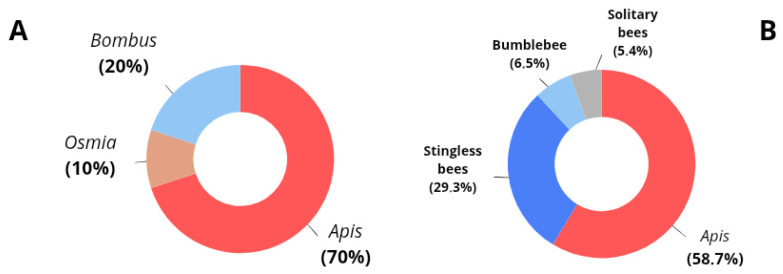
Distribution of test organisms in OECD regulatory documents and in studies on botanical insecticides in bees. (**A**) Distribution of OECD documents according to the organisms covered by test guidelines and guidance documents related to toxicological assessment in bees. (**B**) Relative distribution of records by bee group in studies on botanical insecticides, based on the survey by Catania et al. [22]. In panel B, values represent the proportion of records involving *Apis* spp., stingless bees, bumblebees, and solitary bees in relation to the total number of records by the bee group. OECD documents are not specific to botanical insecticides, but they constitute one of the main standardized references for the toxicological assessment of plant protection products in bees. Data extracted and reorganized from OECD documents [79,80,81,82,83,84,85,86,87,88] and Catania et al. [22]. Figure prepared by the authors.

Reproductive and developmental disturbances represent another critical dimension of sublethal toxicity because they directly affect population persistence. Azadirachtin, due to its insect growth-regulating properties, has been shown to induce deformities in the pupae and adults of *Melipona quadrifasciata* Lepeletier, 1836 [130], as well as developmental delays, morphological abnormalities, and reproductive system atrophy in queens of *Partamona helleri* (Friese, 1900) [96]. Impairment of queen physiology may directly affect oviposition rates and colony growth [131,132], while also compromising the survival of young queens and processes such as queen replacement and colony founding [133,134,135]. In solitary bees, the absence of records in this category in Figure 4 should be interpreted as a lack of targeted studies rather than evidence that these species are unaffected by botanical insecticides [22].

Finally, histological, morphological, physiological, and biochemical alterations represent a less visible dimension of sublethal toxicity and have been reported mainly in *Apis* spp. and stingless bees (Figure 3). Although these cellular, tissue-level, and metabolic impairments may not immediately result in behavioral symptoms or visible mortality, they can compromise essential internal processes, gradually reducing metabolic efficiency and long-term colony survival. Nevertheless, this category remains considerably less explored than lethal and behavioral endpoints, limiting our understanding of the cellular and physiological mechanisms underlying the toxicity of botanical insecticides in bees.

### 4.4. Absence of Detectable Effects of Botanical Insecticides in Bees

Another relevant aspect highlighted in Figure 4 is the occurrence of records classified as “no effect”, indicating the absence of detectable alterations in the evaluated endpoints under the experimental conditions tested. The compilation conducted by Catania et al. [22] identified between 11% and 25% of records without detectable lethal or sublethal effects, depending on the bee group considered. Similarly, reviews focusing on essential oil-based biopesticides report that a substantial proportion of studies found low toxicity or no detectable effects on pollinators [25].

These findings are particularly important because they suggest that certain botanical compounds, formulations, and application rates may be more compatible with pollinators than many conventional synthetic insecticides. Rather than representing a simple absence of biological response, records classified as “no effect” help identify conditions under which botanical insecticides may be used with reduced risk to non-target organisms. Such observations are consistent with characteristics frequently associated with these products, including lower environmental persistence, multiple modes of action, and potential compatibility with Integrated Pest Management (IPM) programs. This pattern becomes even more evident when compared with the extensive body of evidence available for synthetic pesticides. Rossi et al. [8] compiled nearly 200 studies reporting severe lethal and sublethal effects in bees, including impairments in orientation, learning, communication, development, and colony survival. In many cases, mortality approached 100% or resulted in substantial colony losses.

In contrast, Gostin et al. [25] reported that approximately 73% of studies evaluating the lethality of essential oils found mortality rates below 15%, with most values remaining below 4%. Although botanical insecticides can produce adverse effects, the available evidence suggests that these effects are generally less frequent and less severe than those commonly reported for conventional synthetic pesticides. Nevertheless, the absence of detectable effects should not be interpreted as definitive evidence of safety. In some cases, it may reflect methodological limitations, the limited number of available studies, or the lack of assessment of more sensitive endpoints, such as physiological, cognitive, or ecological alterations resulting from sublethal exposure (see Section 5). Therefore, current evidence indicates that botanical insecticides are neither universally safe nor universally harmful to bees. However, when compared with conventional synthetic pesticides, they present a consistent body of evidence supporting their potential as more selective and less disruptive tools for pollinator conservation, provided that their use is guided by appropriate ecotoxicological assessments and Integrated Pest Management principles.

## 5. Limitations of Current Approaches in Selectivity Assessment

The discussion of botanical insecticide selectivity in bees encompasses both the international protocols used in risk assessment and the experimental studies investigating the effects of these products on beneficial organisms. Standardized protocols, such as OECD test guidelines, are essential for guiding regulatory assessments, establishing a common framework for evaluating toxicity in bees, and providing comparable criteria across studies. However, these protocols do not fully encompass pollinator diversity and still present limitations related to experimental settings, exposure routes, life stages evaluated, sublethal effects, and the complexity of formulations and active ingredients found in products such as botanical insecticides.

At the same time, non-regulatory studies have contributed substantially to expanding current knowledge, but they also exhibit an uneven distribution regarding pollinator groups, chemical classes, biological targets, and methodological approaches. When considered together, however, both sources of evidence reveal recurring limitations, particularly those associated with the concentration of experimental models and the difficulty of capturing sublethal responses under realistic field conditions.

### 5.1. Concentration of Experimental Models: Organisms, Life Stages, and Experimental Environments

One of the main limitations of current approaches lies in the concentration of experimental models used to assess the risks of plant protection products to bees. This concentration is evident both in the international regulatory framework established by the OECD and in the independent scientific literature on botanical insecticides, influencing the selection of test organisms, life stages, and experimental settings. Although such standardization enhances reproducibility and comparability among studies, it restricts the reliable extrapolation of findings to the broad diversity of bee species and the multiple exposure pathways encountered in agricultural ecosystems (Figure 4).

**Figure 5:**
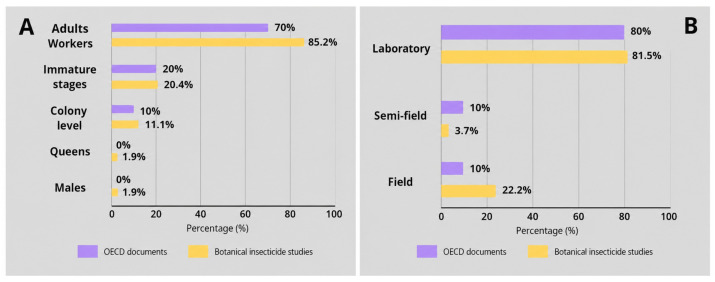
Comparison between OECD regulatory documents and studies evaluating botanical insecticides in bees according to biological target and experimental setting. (**A**) Distribution of OECD documents and botanical insecticide studies according to the life stage or biological target evaluated. (**B**) Distribution according to the experimental setting used in the assays. Values represent the percentage of documents or studies within each category. For studies involving botanical insecticides, categories are not mutually exclusive, as a single study may evaluate more than one biological target or experimental setting. Data extracted and reorganized from OECD documents [79,80,81,82,83,84,85,86,87,88] and Catania et al. [22]. Figure prepared by the authors.

The comparison between OECD documents and the studies compiled by Catania et al. [22] indicate that toxicological assessments in bees remain concentrated on a limited number of biological models. Within the OECD framework, most guidelines are focused on *Apis mellifera* (70%), whereas *Bombus* spp. (20%) and *Osmia* spp. (10%) are represented to a much lesser extent, and no specific documents were identified for stingless bees (Figure 5A). A similar pattern is observed in the scientific literature on botanical insecticides, in which *Apis* spp. account for 58.7% of the records, followed by stingless bees (29.3%), bumblebees (6.5%), and solitary bees (5.4%) (Figure 5B). Although recent years have seen a gradual expansion toward non-*Apis* pollinators, current selectivity assessments still rely heavily on a restricted set of test organisms. As a result, the available evidence may not adequately capture the functional, physiological, and behavioral diversity that characterizes bee communities, limiting the extrapolation of findings across pollinator taxa.

Despite the methodological advantages associated with the use of *A. mellifera* as a surrogate species, its exclusive adoption does not adequately represent the biological diversity of bees [72,135]. Susceptibility to insecticides can vary substantially among species and is influenced by morphological, physiological, and behavioral characteristics [136,137]. This variation is particularly relevant because comparative studies indicate that *A. mellifera* does not always reflect the responses observed in other bee groups. In a systematic review conducted between 2014 and 2025, encompassing 27 studies, 115 experiments, and 24 stingless bee species, these bees were found to be more sensitive to pesticides than *A. mellifera* in 72% of the assays, with adverse effects occurring at lower concentrations [138]. These findings suggest that relying exclusively on *A. mellifera* may underestimate risks to stingless bees and other native pollinators.

However, increased sensitivity should not be interpreted as a universal pattern. Silva et al. (2020) [105], when evaluating ginger, mint, oregano, and thyme essential oils in foragers of *A. mellifera* and *Trigona hyalinata* (Lepeletier, 1836) (Hymenoptera: Apidae), observed that toxicity varied according to the essential oil, application method, and exposure dose. In contrast to the pattern frequently reported for stingless bees, *T. hyalinata* was less susceptible than *A. mellifera* to the tested essential oils. This result reinforces that selectivity should not be considered an intrinsic property of either the insecticide or the bee group, but rather an outcome of the interaction between the organism, the compound, and the exposure conditions.

Differences in susceptibility may be associated with body size, exposure route, tested dose, and physiological mechanisms involved in insecticide metabolism and detoxification [139,140]. Nevertheless, assessing risk solely on the basis of physiological sensitivity remains insufficient, as pesticide exposure is also shaped by ecological traits and landscape context. Characteristics such as sociality, communication systems, colony size, foraging capacity, and dietary breadth [141] directly influence the likelihood of contact with treated crops, contaminated floral resources, and persistent environmental residues.

Thus, species differ not only in how they tolerate insecticides but also in how they encounter them. Bees with extensive foraging ranges, particularly highly eusocial species, may experience greater exposure when exploiting mass-flowering crops and storing contaminated resources for prolonged periods [142]. Conversely, species with more restricted foraging ranges often do not accumulate large resource reserves and therefore depend on continuous resource collection. Under these circumstances, exposure may be reduced when uncontaminated resources are available but may increase substantially in intensively managed agricultural landscapes [143,144]. This perspective is especially important for solitary bees, whose nesting behavior, frequent ground nesting, often univoltine life cycles, and limited foraging ranges may increase vulnerability to residues present in soil, flowers, and other contaminated resources [136,145].

Although risk assessment is gradually moving toward more holistic approaches that incorporate non-*Apis* species and the diversity of wild bees [98,105,130], this progress remains only partially reflected in studies involving botanical insecticides. Consequently, the concentration of assays on *A. mellifera*, adult individuals, and laboratory conditions represents not only a taxonomic or methodological limitation but also an ecological one, restricting our ability to understand how different bee groups are exposed and respond under realistic field conditions. The comparison between OECD documents and studies evaluating botanical insecticides indicates that current limitations in risk assessment are not restricted to regulatory guidelines but are also reflected in the scientific literature itself (Figure 5). In both cases, evaluations remain heavily concentrated on adult workers under laboratory conditions, demonstrating that a large proportion of the available evidence is still derived from biologically simplified scenarios.

Regarding the life stage or biological target evaluated, Figure 5A shows that approximately 70% of OECD documents focus on adult workers, whereas this proportion reaches 85.2% in studies evaluating botanical insecticides. In contrast, immature stages account for 20% of OECD documents and 20.4% of scientific studies, while colony-level assessments represent only 10% and 11.1%, respectively. Queens and males remain largely neglected, appearing in only 1.9% of studies involving botanical insecticides and in none of the OECD documents considered. This distribution indicates that most current inferences regarding safety are still based on responses observed in adult workers. This focus is understandable, as adults are more accessible, easier to manipulate under laboratory conditions, and, in the case of foragers, represent one of the main interfaces between the colony and the external environment. During the collection of pollen, nectar, and water, these individuals may come into direct contact with pesticide residues and subsequently transport them into the colony [136,145].

Nevertheless, the predominance of adult-based assessments provides only a partial representation of real-world exposure scenarios. Larvae and queens may also be exposed through contaminated food, wax, and other materials stored within the colony [66]. Consequently, an exclusive focus on adults may underestimate impacts on brood development, population renewal, and colony reproductive capacity. This limitation is particularly relevant for botanical insecticides that affect growth, feeding, or development. Studies have shown that larval exposure can reduce survival and impair the quality of emerging adults, whereas exposure to azadirachtin during queen development may compromise morphological and reproductive traits associated with colony maintenance [96,97]. Such effects are not adequately captured when assessments are restricted to adult worker survival. Moreover, in *A. mellifera*, queen loss or premature queen replacement is recognized as one of the major factors associated with colony mortality [146]. In solitary bees, this knowledge gap may be even more critical, as developmental or reproductive impairments can directly reduce offspring production, yet these endpoints remain largely unexplored.

Beyond the emphasis on adult bees, Figure 5B reveals another important limitation: the predominance of laboratory-based studies. OECD documents are largely focused on laboratory conditions, which account for 80% of evaluations, and the literature on botanical insecticides follows a similar pattern, with 81.5% of studies conducted under laboratory settings. In comparison, semi-field studies represent 10% of OECD documents and only 3.7% of botanical insecticide studies, whereas field studies account for 10% and 22.2%, respectively. These data indicate that, despite the recognized importance of more realistic exposure scenarios, the available evidence remains strongly dependent on controlled laboratory experiments. These three experimental approaches differ substantially in terms of experimental control and ecological realism.

Laboratory assays are useful for identifying the initial hazard posed by a product because they allow the precise control of dose, exposure route, contact duration, and environmental conditions. However, by relying on standardized exposure scenarios, they often simplify the complexity of bee–pesticide interactions under natural conditions [147]. Semi-field studies represent an intermediate step, maintaining some experimental control while allowing the assessment of responses under more realistic conditions, including foraging activity, contact with floral resources, and early colony-level effects [148]. Field studies provide the highest ecological relevance because they incorporate environmental variables such as volatilization, photodegradation, rainfall, temperature, application frequency, residue persistence, and interactions with other agricultural inputs [23,75,149].

The gap between laboratory and field conditions is particularly important for botanical insecticides, including essential oils and plant extracts, whose chemical stability, volatility, and persistence may vary according to plant species, plant organ, extraction method, and formulation. Consequently, laboratory exposures may overestimate risk when compounds rapidly degrade under field conditions, but they may also underestimate impacts when repeated exposure, contaminated floral resources, interactions among metabolites, or cumulative sublethal effects occur.

**Figure 6:**
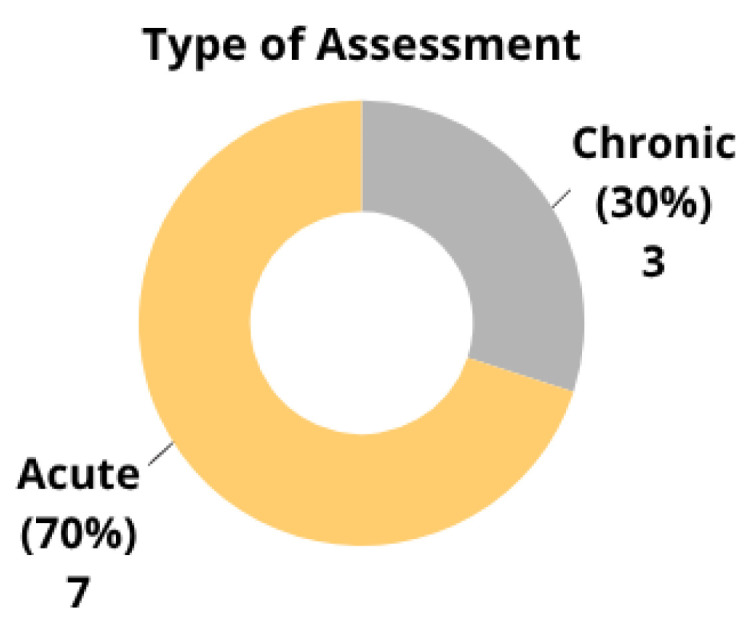
Distribution of OECD documents according to the type of toxicological assessment conducted in bees. The figure highlights the predominance of acute toxicity evaluations, which account for 70% of the OECD documents analyzed, whereas chronic assessments represent 30%. Although these documents are not specific to botanical insecticides, they constitute an important methodological framework for evaluating the effects of plant protection products on bees. Source: OECD [79,80,81,82,83,84,85,86,87,88]. Figure prepared by the authors.

Therefore, selectivity assessments should consider not only where exposure occurs but also its duration and frequency. As discussed in Section 4.3, the absence of mortality does not imply the absence of toxicity. Botanical insecticides may induce sublethal effects across different bee groups, and these responses are not always detected in short-term acute assays. This limitation is also reflected in the OECD framework itself, where approximately 70% of the evaluated guidelines and guidance documents focus on acute toxicity, whereas only 30% incorporate chronic exposure approaches (Figure 6).

Subtle alterations in feeding, locomotion, development, or reproduction may not cause immediate mortality but can progressively impair individual performance and, in social species, colony functioning. This perspective is particularly important for botanical insecticides, as compounds with antifeedant, growth-regulating, or behavioral effects may produce impacts that depend on exposure duration and repeated contact [96,105,129]. Therefore, expanding protocols and studies that incorporate chronic assessments is essential for interpreting the toxicity of botanical insecticides beyond immediate mortality.

In addition to these temporal and environmental limitations, semi-field and field evaluations are important because they allow toxicity to be assessed at more complex levels of biological organization, particularly at the colony level. In social insects, colony size influences sensitivity to environmental stressors because it is associated with the capacity to compensate for individual losses, redistribute tasks, and maintain essential functions such as foraging, brood care, and thermoregulation [150,151]. Although discussing the effects of synthetic insecticides is not the objective of this review, studies involving neonicotinoids—synthetic insecticides derived from nicotine, an alkaloid naturally present in Nicotiana species such as *Nicotiana tabacum* L.—help illustrate this methodological limitation. Rundlöf et al. (2015) [152], for example, observed that under similar exposure conditions, effects were less evident in large *A. mellifera* colonies, intermediate in bumblebees, and more pronounced in solitary bees. Thus, responses to pesticides depend not only on compound toxicity but also on social organization, colony size, and the capacity to buffer individual losses.

This interpretation is particularly relevant for bumblebees, whose colonies are smaller and possess a lower compensatory capacity than those of *A. mellifera*. Exposure during colony foundation, when the colony depends heavily on a single queen, or during early developmental stages may substantially compromise colony performance [153,154]. Consequently, laboratory assessments restricted to adult workers may not adequately represent effects on colony growth, reproduction, and stability. For botanical insecticides, this gap is especially important because products with low acute mortality may still affect essential colony processes through sublethal effects, repeated exposure, or alterations in foraging activity and brood nutrition.

Furthermore, laboratory isolation hinders the evaluation of social interactions that can modify the distribution and intensity of toxic effects within colonies [19]. In eusocial species, processes such as trophallaxis, cooperative brood care, and social immunity may influence how residues are transferred, diluted, or accumulated among individuals. Wueppenhorst et al. [155], for example, demonstrated that nurse bees can function as biological filters through trophallaxis, reducing the transfer of toxic substances present in larval food. Such interactions highlight that the relationship between individual toxicity and colony vulnerability is not always linear. Considering that exposure under field conditions is inherently heterogeneous [156], toxicity assessments should not be limited to a single-exposure route or food source. To better represent realistic exposure scenarios, studies should incorporate multiple routes of exposure, including the ingestion of contaminated resources, contact with environmental residues, and trophallactic transfer, as well as different exposure sources such as nectar, pollen, and water [156,157].

### 5.2. Phytochemical Bias, Mechanistic Gaps, and Risk Mitigation

Finally, the literature on botanical insecticides and bees also presents an important bias regarding the phytochemical classes evaluated. Based on the data compiled by Catania et al. [22], the available evidence is strongly dominated by limonoids, which account for 57.4% of the records, corresponding to 31 studies, and by essential oils, which represent 37.0%, corresponding to 20 studies. In contrast, pyrethrins and phenolic compounds appear less frequently, representing 13.0% and 11.1% of the records, respectively. Other phytochemical classes are even less represented, including alkaloids, with 3.7%, and fatty acids and polyketides, each with 1.9%. This distribution indicates that the current understanding of the effects of botanical insecticides on bees is strongly influenced by a limited number of phytochemical groups, while several bioactive classes remain poorly investigated.

This distribution does not necessarily indicate that the less-studied classes are safer, but it reveals an important limitation in the mechanistic interpretation of selectivity. Because different phytochemical classes exhibit distinct modes of action, the concentration of studies on a limited number of groups reduces our ability to compare risks among active substances and identify compounds that may pose greater risks to pollinators. Furthermore, the available evidence remains insufficient to represent the full chemical diversity of plant secondary metabolites, limiting broader conclusions regarding the selectivity and safety of botanical insecticides in bees [158].

Catania et al. [22] highlighted that botanical insecticides encompass classes such as alkaloids, essential oils, fatty acids, limonoids, phenolic compounds, polyketides, and pyrethrins, each possessing potentially distinct chemical properties and modes of action. Among the most extensively studied classes, limonoids, particularly azadirachtin-based products derived from *A. indica*, provide the most consistent body of toxicological evidence in bees. Their effects are frequently associated with developmental and reproductive impairments due to their insect growth-regulating properties [96,130,159,160,161]. These mechanisms help explain why azadirachtin may produce more pronounced effects following oral exposure and in immature stages, queens, or microcolonies, even when adult mortality remains relatively low [22,96,130,160]. Therefore, the risk posed by limonoids should not be assessed solely through acute mortality but also through their capacity to affect feeding, metamorphosis, reproductive development, and colony maintenance.

Essential oils represent another important group, although their toxicological interpretation is considerably more complex. As mixtures of volatile compounds, frequently rich in terpenoids such as monoterpenes and sesquiterpenes, as well as phenolic constituents, these products do not possess a fixed composition and may vary substantially according to plant species, plant organ, extraction method, formulation, and concentration. Such chemical variability directly influences the toxicity profile observed in bees. In general, the effects associated with essential oils have been primarily linked to behavioral and functional alterations, including repellency, reduced locomotion, impaired orientation, and decreased foraging activity [162,163,164,165,166,167,168]. However, the magnitude of these effects depends on the predominant chemical composition of the oil, the exposure route, and the experimental conditions. This variability helps explain why some essential oils exhibit low toxicity under certain conditions, whereas others induce mortality, repellency, locomotor impairment, or reductions in foraging activity [22,97,105]. Consequently, essential oils should not be treated as a uniform toxicological category, as their risk to pollinators depends on both product composition and exposure scenario.

The less represented phytochemical classes are particularly important because they highlight a major mechanistic gap in the literature. Alkaloids, for example, exhibit strong insecticidal activity at low doses and may also induce sublethal effects, including feeding impairment, developmental abnormalities, and stimulatory responses [22,169,170,171]. Nevertheless, these compounds remain poorly investigated, particularly in non-*Apis* bee groups [22]. Thymol, a compound commonly found in essential oils, may display relatively low lethal toxicity under certain conditions while still inducing behavioral responses such as avoidance in stingless bees. Conversely, compounds such as rotenone have been associated with both lethal and sublethal effects in *A. mellifera*, including locomotor alterations [22,172]. Pyrethrins represent another underrepresented class in studies evaluating botanical insecticides in bees, despite their well-established neurotoxic mode of action. Derived from *Tanacetum cinerariifolium* (Trevir.) Sch.Bip., these plant-based compounds interfere with nerve impulse transmission and may induce paralysis. However, the limited availability of recent studies evaluating botanical pyrethrins in different bee groups restricts our ability to assess the risks associated with this class [173].

Thus, the primary limitation is not merely the low frequency of studies involving certain phytochemical classes, but rather the mismatch between chemical diversity and mechanistic understanding. The available literature allows a relatively robust discussion of limonoids and, to a lesser extent, essential oils, yet provides little basis for determining whether alkaloids, pyrethrins, phenolics, fatty acids, and polyketides are genuinely less toxic to bees or simply underexplored. This limitation is further exacerbated when studies fail to adequately characterize extract composition, identify major constituents, include positive controls, or select endpoints consistent with the expected mode of action.

Understanding these mechanisms is essential because similar ecological outcomes may arise from distinct physiological processes, leading to different long-term consequences for colony performance and pollination services. In this context, although most mitigation strategies currently employed were originally developed for synthetic pesticides, their underlying principles remain applicable to botanical insecticides. However, the widespread perception that plant-derived products are inherently safe may lead to an underestimation of the importance of such measures. Therefore, mitigation strategies already established for pollinator protection should also be considered when using botanical insecticides, particularly when evidence indicates the occurrence of lethal and sublethal effects in bees.

Mitigating the risks posed by pesticides with systemic activity requires careful management of chronic exposure. Although limonoids such as azadirachtin act primarily as insect growth regulators, their residual persistence may affect larval viability and queen ovarian morphometry when transported by nurse workers [160]. Under these circumstances, applications should be avoided during crop flowering periods and during the blooming of adjacent spontaneous vegetation. In addition, refuge areas containing native vegetation should be maintained within a radius of up to 1.5 km, providing floral resources free from persistent residues [174].

For compounds that interfere with neural and sensory functions, mitigation should focus on managing residual activity and formulation characteristics in order to reduce disorientation and locomotor disturbances in bees. These insecticides may impair motor coordination, olfactory memory, and the accuracy of social communication mechanisms [8]. As a preventive measure, applications should preferably be conducted during twilight or nighttime periods, allowing photolabile compounds such as pyrethrins to degrade before peak foraging activity. Furthermore, granular or solution-based formulations may represent safer alternatives than wettable powders or microencapsulated formulations, whose particles can adhere to bee body hairs and facilitate both contact exposure and residue transport into the colony [175].

From a practical perspective, mitigation strategies should accompany toxicological assessments. Measures such as avoiding applications during flowering, conducting sprays outside peak foraging periods, reducing field application rates whenever agronomically feasible, and prioritizing compounds with lower toxicity to pollinators may substantially reduce exposure. In addition, advances in formulation technologies may improve selectivity by reducing environmental persistence and limiting contact with non-target organisms. Incorporating these strategies into Integrated Pest Management programs may maximize the benefits of botanical insecticides while minimizing risks to pollinator communities.

## 6. Future Perspectives

Future perspectives for the use of botanical insecticides within Integrated Pest Management (IPM) require a profound reassessment of the safety paradigm traditionally associated with these compounds in relation to bees. Although interest in these biopesticides has increased due to the demand for less persistent alternatives compatible with resistance management, the assumption that their natural origin guarantees intrinsic selectivity has been challenged by growing scientific evidence. Future research in this field should therefore focus on deconstructing this concept of “automatic safety”, subjecting plant secondary metabolites to the same toxicological scrutiny applied to synthetic molecules.

A critical advance required in the coming decades is the expansion of biological diversity in ecotoxicological studies, reducing the excessive dependence on *A. mellifera* as the sole surrogate species. Currently, approximately 70% of OECD documents are centered on this species, leaving substantial gaps regarding impacts on solitary bees, bumblebees, and especially stingless bees of the tribe Meliponini (Figure 4A). Future perspectives point to the urgent need for the development of official protocols specifically designed for stingless bees, considering that these species may exhibit greater sensitivity than *A. mellifera* under several exposure scenarios and still lack standardized testing guidelines [138,176].

In addition, risk assessment must evolve beyond the analysis of acute mortality, prioritizing sublethal and chronic effects capable of compromising colony viability in the long term. Future studies should standardize the evaluation of essential functions such as foraging ability, spatial orientation, and learning, which are crucial for colony survival. Investigations should also systematically include immature stages and reproductive castes, such as queens and drones, whose physiological and reproductive impairments are often neglected in conventional toxicity assays despite their importance for population maintenance.

Another major challenge for future research is increasing environmental realism in experimental assays. The current predominance of laboratory studies, which account for approximately 81.5% of available evaluations, should be balanced with a greater number of semi-field and field studies (Figure 5B). This shift would allow researchers to better capture the influence of factors such as photodegradation, residue persistence, and complex social interactions, including trophallaxis, which may either mitigate or intensify pesticide risks. Likewise, understanding the chemical complexity of botanical products, which function as multicomponent mixtures, represents a promising area for future discoveries involving synergistic interactions among metabolites and their interactions with other agrochemicals present in agricultural landscapes.

The chemical complexity of botanical insecticides simultaneously represents both a challenge and an opportunity for future research. Unlike many synthetic pesticides based on isolated active ingredients, plant extracts and essential oils consist of multicomponent mixtures with possible synergistic or antagonistic interactions. Although this complexity may reduce the probability of resistance evolution in pest insects, it also complicates toxicological interpretation and experimental standardization. Therefore, future studies should prioritize the characterization of chemical composition, formulation stability, degradation dynamics, and interactions with other agrochemicals frequently used in agricultural systems.

Within this context, it becomes evident that protocols originally developed for isolated synthetic molecules may not be sufficient for the proper evaluation of botanical insecticides, whose chemical composition may vary according to plant species, plant organ, extraction method, seasonality, and formulation. Moreover, the effects of these mixtures cannot always be attributed solely to the major compound, since secondary metabolites may act synergistically, altering toxicity, persistence, and sublethal effects in non-target organisms. Consequently, the development of specific protocols for botanical insecticides is necessary, taking into account not only their chemical complexity, stability, and environmental degradation, but also their lethal and sublethal effects across different bee groups.

Finally, international regulatory harmonization emerges as an imperative need to ensure that the expansion of the botanical insecticide market does not occur at the expense of biodiversity. The development of guidelines incorporating more complex behavioral and physiological endpoints will enable the safer integration of these biopesticides into sustainable agricultural programs, effectively protecting pollination ecosystem services and contributing to global food security.

## 7. Conclusions

Although botanical insecticides are frequently associated with the concept of selectivity, they exhibit a broad diversity of mechanisms of action capable of affecting bees at multiple biological levels. Beyond acute toxicity, sublethal effects—including behavioral, physiological, and developmental alterations—may compromise essential functions such as foraging and reproduction. This scenario reflects a historical mismatch between the expansion in the use of these compounds and the assessment of their impacts on non-target organisms, resulting in important gaps in the understanding of their ecological safety.

From methodological and regulatory perspectives, current protocols remain limited in their ability to capture this complexity due to the predominance of laboratory assays, the strong focus on *A. mellifera*, and the low standardization among studies. In addition, the chemical complexity of botanical insecticides hinders broad generalizations regarding their effects. Therefore, more integrative approaches are needed, considering different mechanisms of action, species, and ecological contexts, in order to critically reassess the selectivity of these compounds and support the development of safer pest management strategies for pollinators.

## Figures and Tables

**Table 1:** OECD guidelines and guidance documents applicable to the toxicological assessment of plant protection products in bees. The table summarizes the main test guidelines (TGs) and guidance documents (GDs), including the respective test organisms, experimental settings, exposure routes, and evaluated endpoints. These protocols are not specific to botanical insecticides but are currently used as the primary framework for assessing the potential effects of plant protection products on pollinators. Source: OECD [79,80,81,82,83,84,85,86,87,88].

OECD Document	Type	Organism	Environment	Exposure	Main Focus
TG 213—Honeybees, Acute Oral Toxicity Test	Test Guideline (TG)	Adult *Apis mellifera* workers	Laboratory	Oral, via contaminated sucrose solution	Acute oral toxicity; mortality; LD50 at 24/48 h, extending up to 96 h.
TG 214—Honeybees, Acute Contact Toxicity Test	Test Guideline (TG)	Adult *A. mellifera* workers	Laboratory	Topical contact, application on the thorax	Acute contact toxicity; mortality; LD50 at 24/48 h, extending up to 96 h.
TG 237—Honey Bee Larval Toxicity Test, Single Exposure	Test Guideline (TG)	*A. mellifera* larvae	Laboratory	Single exposure via artificial diet	Acute larval toxicity; larval mortality and LD50 at 72 h.
TG 245—Honey Bee Chronic Oral Toxicity Test, 10-Day Feeding	Test Guideline (TG)	Young adult *A. mellifera* workers	Laboratory	Chronic oral, feeding with contaminated sucrose for 10 days	Chronic oral toxicity; mortality, behavioral abnormalities, LC50, LDD50, NOEC, and NOEDD.
TG 246—Bumblebee, Acute Contact Toxicity Test	Test Guideline (TG)	Adult workers of *Bombus* spp., especially *Bombus terrestris* and *Bombus impatiens*	Laboratory	Topical contact on the dorsal thorax	Acute contact toxicity; mortality; LD50 and NOED, where possible.
TG 247—Bumblebee, Acute Oral Toxicity Test	Test Guideline (TG)	Adult workers of *Bombus* spp., especially *Bombus terrestris* and *Bombus impatiens*	Laboratory	Oral, via contaminated 50% sucrose solution	Acute oral toxicity; mortality; LD50 and NOED, where possible.
TG 254—Mason bees (*Osmia* sp.), Acute Contact Toxicity Test	Test Guideline (TG)	Adult females of solitary bees of the genus *Osmia*	Laboratory	Topical contact on the dorsal thorax	Acute contact toxicity; mortality; LD50 at 48 h, extending up to 96 h; observable sublethal effects should also be recorded.
GD 75—Guidance Document on the Honey Bee Brood Test Under Semi-Field Conditions	Guidance Document (GD)	*A. mellifera* colonies/nuclei	Semi-field (tunnels), followed by field conditions	Exposure of the colony/brood to the product applied to agricultural crops	Quantitative assessment of adverse effects on brood development; especially relevant for growth regulators and larvicidal compounds. The first edition was published as No. 75; the second edition was published in 2024.
GD 239—Guidance Document on Honey Bee Larval Toxicity Test following Repeated Exposure	Guidance Document (GD)	*A. mellifera* larvae	Laboratory	Repeated exposure via artificial diet	Larval toxicity by repeated exposure; larval mortality, pupal mortality, adult emergence, NOEC/NOED and, if possible, EC50/ED50.
GD 332—Guidance Document on Honey Bee Homing Flight Test	Guidance Document (GD)	*A. mellifera* foragers	Semi-field/Field	Single sublethal oral exposure in the laboratory, marking, and release in the field	Sublethal effect on orientation and homing success after oral exposure; evaluates return flight success under short-term conditions closer to the field.

## Data Availability

No new data were created or analyzed in this study.
